# The nucleus of endothelial cell as a sensor of blood flow direction

**DOI:** 10.1242/bio.20134622

**Published:** 2013-08-14

**Authors:** Eugene Tkachenko, Edgar Gutierrez, Semion K. Saikin, Per Fogelstrand, Chungho Kim, Alex Groisman, Mark H. Ginsberg

**Affiliations:** 1 Department of Medicine, University of California-San Diego, La Jolla, CA 92093, USA; 2 Department of Physics, University of California-San Diego, La Jolla, CA 92093, USA; 3 Department of Chemistry and Chemical Biology, Harvard University, Cambridge, MA 02138, USA; 4 Department of Molecular and Clinical Medicine, Gothenburg University, SE-413 45 Gothenburg, Sweden

**Keywords:** Mechanotransduction, Planar cell polarity, Endothelium, Shear stress, Nucleus

## Abstract

Hemodynamic shear stresses cause endothelial cells (ECs) to polarize in the plane of the flow. Paradoxically, under strong shear flows, ECs disassemble their primary cilia, common sensors of shear, and thus must use an alternative mechanism of sensing the strength and direction of flow. In our experiments in microfluidic perfusion chambers, confluent ECs developed planar cell polarity at a rate proportional to the shear stress. The location of Golgi apparatus and microtubule organizing center was biased to the upstream side of the nucleus, i.e. the ECs polarized against the flow. These in vitro results agreed with observations in murine blood vessels, where EC polarization against the flow was stronger in high flow arteries than in veins. Once established, flow-induced polarization persisted over long time intervals without external shear. Transient destabilization of acto-myosin cytoskeleton by inhibition of myosin II or depolymerization of actin promoted polarization of EC against the flow, indicating that an intact acto-myosin cytoskeleton resists flow-induced polarization. These results suggested that polarization was induced by mechanical displacement of EC nuclei downstream under the hydrodynamic drag. This hypothesis was confirmed by the observation that acute application of a large hydrodynamic force to ECs resulted in an immediate downstream displacement of nuclei and was sufficient to induce persistent polarization. Taken together, our data indicate that ECs can sense the direction and strength of blood flow through the hydrodynamic drag applied to their nuclei.

## Introduction

Most tissues are characterized by coherent cellular polarization: individual cells have opposing sides with distinct properties and the orientations of imaginary axes connecting these sides are highly coordinated (Simons and Mlodzik, 2008). This coordination of the polarization axes between cells in the tissue is critical for development and function of organs (Simons and Mlodzik, 2008), and is referred to as planar cell polarity (Sepich et al., 2011). In the case of planar cell arrangements, the two opposing sides of the cell are the back, where the nucleus is located, and the front, with the Golgi apparatus, centrosomes and the Microtubule Organization Center (MTOC). During polarization, acto-myosin cytoskeleton was shown to be required for relocation of the nucleus (Folker et al., 2011; Gomes et al., 2005; Gundersen and Worman, 2013) and MTOC (Etienne-Manneville and Hall, 2001; Morgan et al., 2011; Tzima et al., 2003). In many tissues, particularly in endothelium, planar cell polarity develops in response to shear flow (McCue et al., 2006; Morgan et al., 2011; Tzima et al., 2003).

Studies on flow-induced planar cell polarity of ECs provided somewhat conflicting data on whether EC polarization, as described by the orientation of vectors drawn from the back to the front of ECs, is along or against the flow (Galbraith et al., 1998; Gotlieb et al., 1981; Kiosses et al., 1997b; McCue et al., 2006; Morgan et al., 2011). Observed discrepancies in the direction of polarization were attributed to vasculature type-specific and age-related differences in gene expression of ECs (McCue et al., 2006). One of the factors promoting polarization of ECs against the flow is a low level of activity of GSK-3β (McCue et al., 2006). GSK-3β activity is known to lead to destabilization of β-catenin in cell junctions (Castelo-Branco et al., 2004) and to stabilization of microtubules (McCue et al., 2006), resulting in reduction of the turnover rate of focal adhesions (Kaverina et al., 1999). In addition to GSK-3β activity, polarization of ECs under shear is known to be directly affected by the state of cell junctions. For example, sparsely seeded ECs in vitro are polarized along the flow (Zaidel-Bar et al., 2005), whereas wounding of endothelial monolayer causes predominant planar cell polarity toward the wound independent of the direction of the flow (Li et al., 2005; Tkachenko et al., 2009). Disruption of cell junction-localized endothelial-specific VE-cadherin/VEGFR2/PECAM-1 mechanosensing complex by silencing any component of this complex inhibits flow-induced actin cytoskeleton rearrangement and cell elongation along the flow (Tzima et al., 2005) that normally accompanies the development of planar polarity. Altogether, these data indicate that the state of cell junctions and dynamic rearrangements of cytoskeleton are important for the establishment of flow-induced planar cell polarity of endothelium (Conway and Schwartz, 2012).

Many types of cells are known to sense the flow by the primary cilium (Simons and Mlodzik, 2008), which becomes increasingly tilted as the shear stress grows, resulting in stronger cellular responses to greater shear stresses. However, it remains unclear, how the deformation of cilia is translated by the cell into a cue about the direction (rather than strength) of the flow, allowing the cell to align its polarization relative to the flow. Moreover, planar cell polarity is exhibited by ECs under strong shear flows (Kiosses et al., 1997b; McCue et al., 2006; Rogers et al., 1985) when most of them do not possess primary cilium (Iomini et al., 2004; Van der Heiden et al., 2006). Therefore, ECs are likely to be using a mechanism of sensing the direction of blood flow that does not rely on primary cilia.

EC could sense the direction of flow with some sensory structures (or structure), which are shifted towards the downstream side of the cell under the action of shear stress (Davies and Tripathi, 1993). Such sensory structures have never been identified, however. Here we report experiments in microfluidic perfusion chambers and observations in vivo, suggesting that the role of a structure sensing the direction of blood flow in ECs can be played by the nucleus, which is pushed downstream by the hydrodynamic drag.

## Results

### Hydrodynamic stress induces polarization of endothelium against the flow

**Fig. 1.**
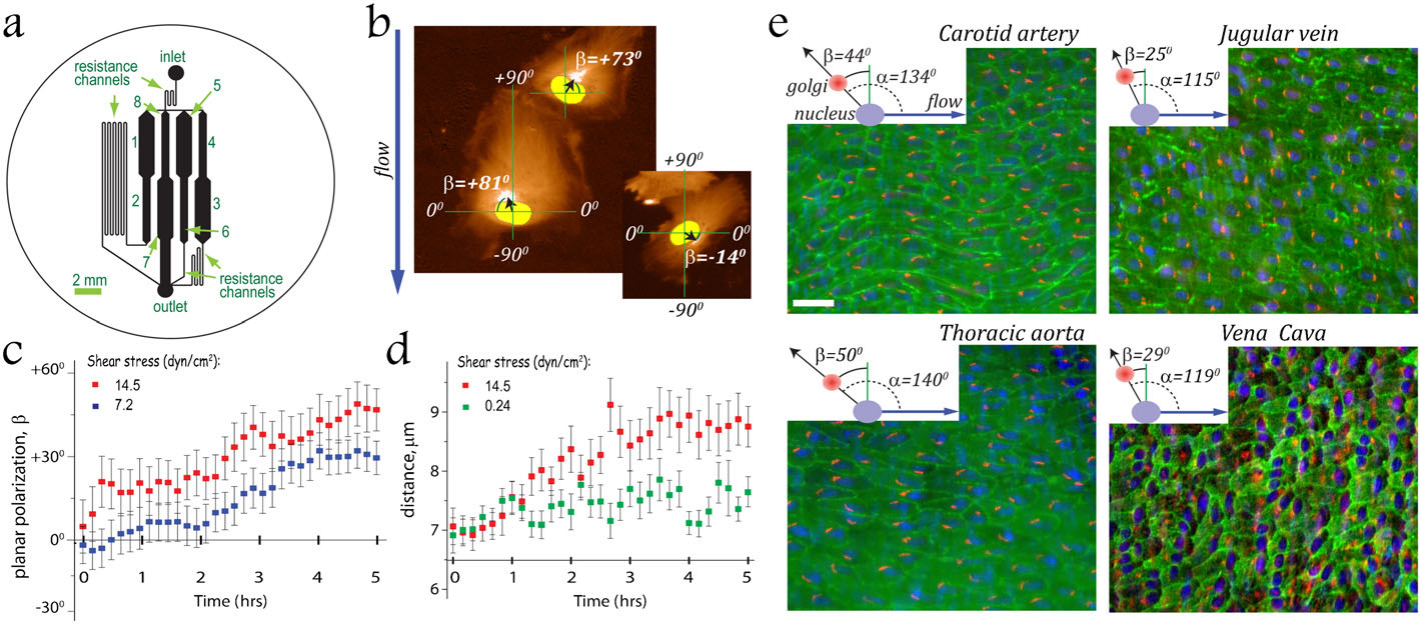
Hydrodynamic stress induces polarization of endothelium against the flow. (a) The microfluidic device was designed to have 2-fold variation of shear stresses between test regions with consecutive numbers, and a total 128-fold range in shear stresses. Numbers 1–8 indicate different test regions that are 1.2 and 0.6 mm wide rectangular channels. Depth of channels is 75 µm. (b) The degree of cell polarization with respect to the flow direction was scored by drawing a cell polarization vector from the center of H2b–mCherry labeled nucleus (yellow) to the center of MTOC [the brightest signal (*white*) from GFP–α-tubulin (*brown*)], measuring the angle *α* between this vector and the vector of flow velocity (*blue arrow*), and calculating a polarization angle *β* = *α*−90°, with *β* = 90° (and *α* = 180°) corresponding to perfect polarization against the flow. (c,d) Abscissa corresponds to time after the inception of flow with a shear stresses *τ* = 14.5 (*red*, *n* = 46, 3 exp.), 7.2 (*blue*, *n* = 29, 3 exp.) and 0.24 (green, *n* = 28, 3 exp.) dyn/cm^2^. Error bars are SEM. (****c) The average degree of cell polarization, mean *β*, as a function of time. (d) The average distance between the centers of the nucleus and MTOC as a function of time. (e) Polarization of ECs in mouse vessels. The planar polarity of EC with respect to the direction of blood flow was scored by drawing a cell polarization vector from the center of the nucleus (*blue*) and to that of the Golgi (*red*), measuring the angle *α* between this vector and the vector of flow velocity (*blue arrow*), and calculating *β* = *α*−90°. Inserts indicate average *α* and *β* in carotid artery (SEM = 2.6°, *n* = 317, 3 mice), thoracic aorta (SEM = 1.4°, *n* = 857, 2 mice), jugular vein (SEM = 3.9°, *n* = 157, 2 mice) and inferior vena cava (SEM = 2.6°, *n* = 424, 2 mice). *Green* depicts an expression of endothelial marker PECAM-1. Scale bar: 30 µm.

To examine changes in planar polarity of ECs in response to hydrodynamic forces, confluent Human Umbilical Vein Endothelial Cells (HUVECs) plated on coverslips coated with human fibronectin were exposed to shear stress, *τ*, ranging from 0.12 to 14.5 dyn/cm^2^ in a microfluidic device (Fig. 1a) (Tkachenko et al., 2009). Application of high shear stresses gradually made HUVECs more elongated and lead to preferred orientation of their major axes along the direction of flow, whereas at low shear stresses HUVECs did not become visibly elongated and their orientation was random (supplementary material Fig. S1). This alignment of HUVECs parallel to the direction of high shear flows agreed with previous reports (Levesque and Nerem, 1985; Tkachenko et al., 2009; Tzima et al., 2005). Shear stresses that resulted in cellular alignment also lead to polarization of HUVECs against the flow (Morgan et al., 2011). To quantify the polarization, we drew a vector from the center of the nucleus to the center of the MTOC (Fig. 1b) or the Golgi apparatus (supplementary material Movie 1), measured the angle *α* that this vector formed with the direction of flow (0°–180°) and calculated a polarization angle *β* = *α*–90°. A perfect polarization of a cell against the flow corresponds to *α* = 180° and *β* = 90°, whereas a random polarization of cells results in *α* randomly varying between 0 and 180° and in *β* = 0° on average. Under shear stresses ≥7.2 dynes/cm^2^, the Golgi apparatus and MTOC gradually became situated upstream of the nucleus (supplementary material Movies 1, 2) and the average polarization angle, *β*, gradually increased (Fig. 1c). The eventual level of polarization, as measured by the value of *β*, and the rate of its establishment both increased with *τ* (the former increase was in agreement with previous reports) (Galbraith et al., 1998) (Fig. 1c). Increase in level of polarization correlated with increase in the average distance between the nucleus and MTOC (Fig. 1d), probably due to elongation of HUVECs. The high level of polarization achieved at large *τ* persisted for at least 1 hr after the flow was stopped.

We also tested the EC polarization in vivo, in large vessels of 10 week old mice. ECs were preferentially polarized against the flow in the jugular vein, with *β* = 25°±3.9° (mean ± SEM; *n* = 157, 2 mice), and inferior vena cava, with *β* = 29°±2.6° (*n* = 424, 2 mice) (Fig. 1e). These findings differ from the reported preferential polarization of venous ECs along the flow in large vessels of pigs and rabbits (McCue et al., 2006; Rogers et al., 1985) that might be due to age-dependent differences in the polarization of EC in response to flow (discussed by McCue et al., 2006). Likewise, we observed preferential polarization against the flow of mouse ECs in the carotid artery (*β* = 44°±2.6°, *n* = 317, 3 mice) and thoracic aorta (*β* = 50°±1.4°, *n* = 857, 2 mice) (Fig. 1e), in agreement with previous reports in rats, pigs and rabbits (Kiosses et al., 1997a; Rogers et al., 1985).

### Formation of a dense lamellum between the nucleus and leading edge in non-confluent ECs

**Fig. 2.**
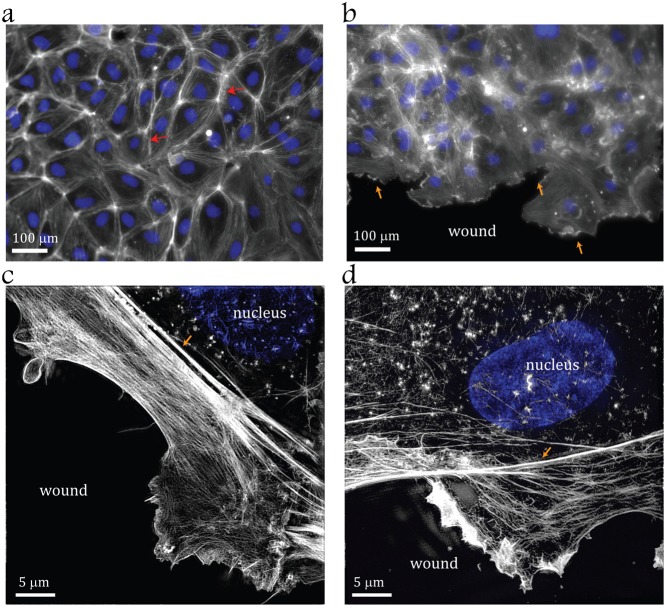
Formation of a dense lamellum between the nucleus and leading edge in non-confluent endothelial cells. F-actin (*grey*) and nuclei (*blue*) were stained in confluent (a) and scratch wounded (b,c,d) HUVECs. (a) *Red arrows* indicate cortical actin structures. (b) *Orange arrows* indicate the leading edge. (c) Super resolution images of actin structures at the leading edge (*orange arrow*). (d) Super resolution images of actin structures at the leading edge (*orange arrow*) of HUVEC on the upstream edge of the wound after 30 min exposure to shear stress *τ* = 11 dyn/cm^2^. Scale bars: 100 µm (a,b), 5 µm (c,d).

Previous in vitro studies reported polarization along the flow on the upstream side of a wounded EC monolayer (Li et al., 2005; Tkachenko et al., 2009), whereas we have now observed that confluent cells polarize against the flow, i.e. in the opposite direction (Fig. 1d). These two observations suggest the importance of cell-to-cell contacts in dictating the sense of EC polarization in response to hydrodynamic stress. Polarization of ECs is established by displacement of the nucleus with respect to MTOC and Golgi apparatus and this displacement is likely to be influenced by the actin-myosin-II cytoskeleton (Gomes et al., 2005; Tzima et al., 2003). Therefore, we examined actin architectures of ECs in confluent and scratch-wounded EC monolayers using confocal and super resolution microscopy. Confluent HUVECs exhibited pronounced actin cortical bundles at cell-to-cell contacts (Fig. 2a). Wounding of endothelial monolayer resulted in major rearrangements of actin cytoskeleton, as indicated by disappearance of cortical F-actin bundles and formation of a lamellipodium at the wound edge (Fig. 2b). Between the lamellipodium and bulk cell body, a dense actin cytoskeleton mesh bundled by myosin II forms a morphologically distinct cellular region termed the lamellum (Fig. 2c) (Ponti et al., 2004) that is preserved after application of shear flow (Fig. 2d).

### The actin cytoskeleton blocks polarization against the flow in non-confluent EC

**Fig. 3.**
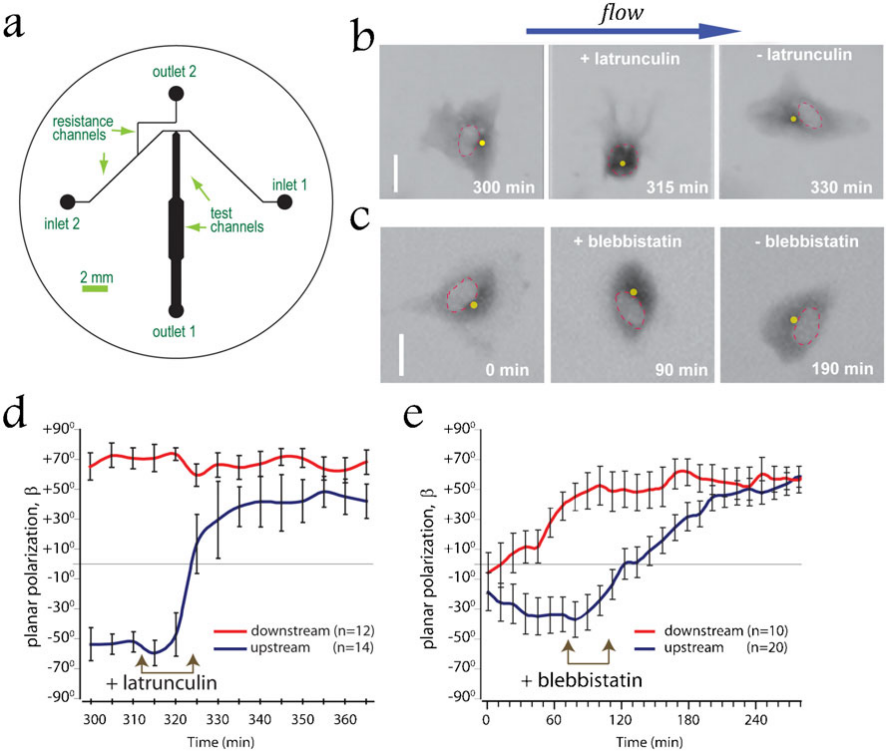
The actin cytoskeleton blocks polarization against the flow in non-confluent endothelial cells. (a) The device designed for controlled addition of pharmacological reagents without changing shear applied to ECs. Plain medium is fed from inlet 1 (normally open); medium with reagent is fed from inlet 2 (normally blocked); outlet 2 (normally open) is used to fill the device, such that a reagent can be rapidly applied when needed, but without its premature leaking into the test channels. Inlets 1 and 2 are equally pressurized, so flow of media with and without a reagent are at the same shear stress. All channels are 73 µm deep. (b,c) Rapid change of the relative positioning of the nucleus and the MTOC in HUVEC exposed to shear stress *τ* = 7.2 dyn/cm^2^ after transient depolymerization of F-actin due to the treatment with 1 µM latrunculin A for 15 min (b) or after inhibition of myosin II due to the treatment with 30 µM blebbistatin for 30 min (c). Inversed fluorescent signal of GFP–α-tubulin is depicted in *grey*. *Red dashed lines* outline nuclei. *Yellow dots* indicate position of MTOCs. Flow is directed from left to right (*blue arrow*). Scale bars: 20 µm. (d,e) Mean degree of cell polarization, *β*, as a function of time for cells along the upstream (*blue*) and downstream (*red*) sides of the wound. Wounded HUVECs monolayer was exposed to shear stress *τ* = 7.2 dyn/cm^2^ for 310 min (d) or 70 min (e) followed by transient application of 1 µM of latrunculin A for 15 min (d) or blebbistatin for 30 min (e). Error bars are SEM.

The polarization of ECs in the direction of flow at the upstream side of the wound in a wounded monolayer may be due to the fact that rearward displacement of their nuclei is prevented by dense actin cytoskeleton mesh of lamellum (Fig. 2d) (Li et al., 2005). To test this hypothesis we altered the structure of actin cytoskeleton by two types of treatments. Treatment of the wounded monolayer with latrunculin A led to nearly complete disassembly of all F-actin structures, whereas inhibition of myosin II by blebbistatin resulted in thinning of actin cables in the lamellum region (supplementary material Fig. S2). These treatments were applied to a monolayer of HUVECs with a scratch wound in a microfluidic perfusion chamber under a continuous flow with a shear stress of 7.2 dyn/cm^2^. The direction of the flow was perpendicular to the wound (Fig. 3a), and the perfusion chamber had two inlets for alternative injection of different media. As anticipated, untreated cells on the upstream side of the wound were polarized along the flow (MTOC located downstream of the nucleus and *β*<0). Transient treatment with latrunculin A (Fig. 3b; supplementary material Movie 3) or blebbistatin (Fig. 3c; supplementary material Movie 4) caused relocation of nuclei to positions downstream of the MTOC, leading to a reversal of polarity in the cells on the upstream side of the wound and to average *β*>0 (Fig. 3d,e). Thus, transient destabilization of actin cytoskeleton by depolymerization of F-actin or inhibition of myosin-II promotes polarization of HUVECs against the direction of flow. Notably, prolonged (>30 min) exposure of HUVECs to blebbistatin under a shear stress of 7.2 dyn/cm^2^ lead to rupture of the plasma membrane and detachment of nuclei from cells, providing further evidence for substantial hydrodynamic drag experienced by HUVEC nuclei under the shear flow.

### Hydrodynamic drag mechanically displaces the nucleus downstream, inducing planar polarization of ECs

In EC, the nucleus creates a bulge on the apical cell surface (Barbee et al., 1994; Hazel and Pedley, 2000). The distribution of hydrodynamic stresses around the cell in shear flow generates a net force pushing this bulge (and the nucleus under the bulge) downstream (Wang and Dimitrakopoulos, 2006). The nuclear envelope is connected to actin cytoskeleton (Gomes et al., 2005), but those connections are dynamic, as indicated by tubulin motor-driven rotations of the nucleus (Levy and Holzbaur, 2008). Therefore, we suggested that the observed polarization of ECs under shear flow can be purely mechanical, directly induced by hydrodynamic drag applied to their nuclei. Under the action of the hydrodynamic drag, the cytoskeleton gradually rearranges, allowing for a slow downstream drift of the nucleus. The rate of the drift can then be expected to increase with shear stress (that the hydrodynamic drag is proportional to), leading to faster polarization at higher shear stress, as was indeed observed in our experiments (Fig. 1d). Furthermore, weakening of the actin cytoskeleton is expected to lead to reduced intracellular resistance to the downstream drift of the nucleus under shear, thus resulting in rapid polarization of ECs against the flow, also in agreement with our results (Fig. 3d,e). The observed persistence of EC polarization after the flow has been stopped is also consistent with the proposed polarization mechanism, because the persistence indicates the absence of rapid-action cellular mechanisms actively restoring ECs to non-polarized states, when no external mechanical cues are applied.

**Fig. 4.**
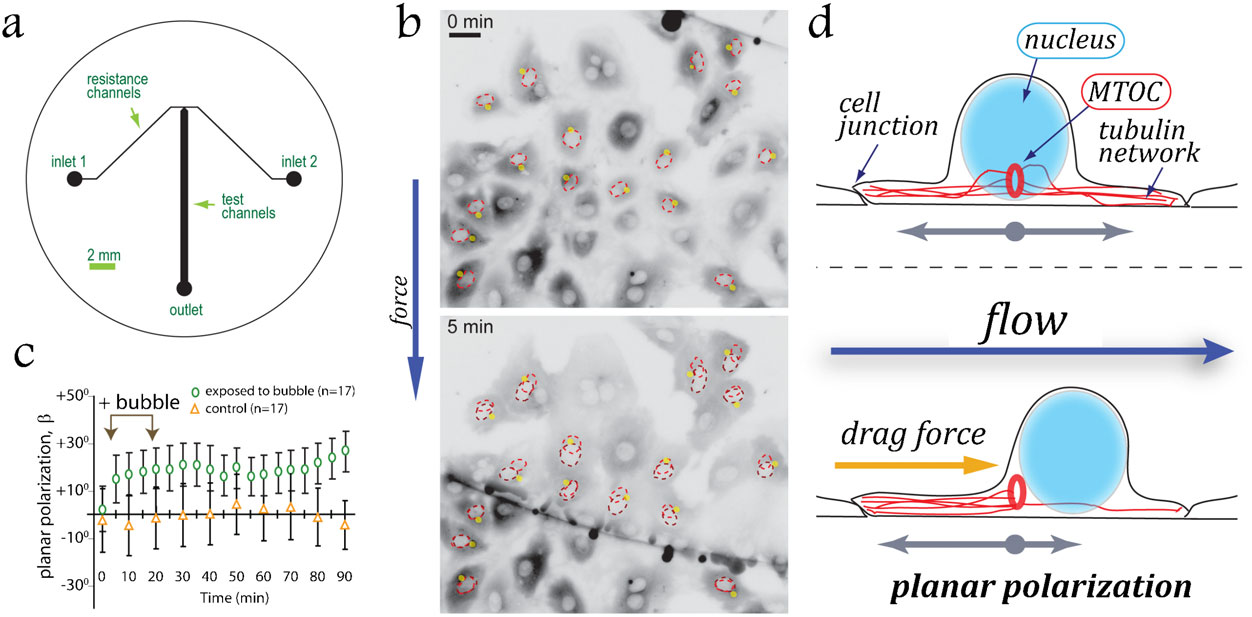
Hydrodynamic drag mechanically displaces the nucleus downstream, inducing planar polarization. HUVECs were exposed to a short-term hydrodynamic drag from a passing air bubble. (a) The device was designed to introduce an air bubble into microchannels seeded with cells. A constant pressure is maintained at *inlet 1* resulting in a shear stress *τ* = 0.14 dyn/cm^2^ in the test channel. *Inlet 2* is fed by pressure regulated compressed air, which is used to form a bubble. This bubble invades into test channel and is pinched off by depressurizing the air. All channels are 75 µm deep. (b) Mechanical displacements of nuclei in HUVECs under an advancing air bubble (*boundary seen as a black line*). Direction of the air bubble passage is from top to bottom. *Dashed outline* shows the positions of nuclei before (*red*) and after (*brown*) passage of the bubble. Inversed fluorescent signal of GFP–α-tubulin is depicted in *grey*. *Yellow dots* indicate positions of MTOCs. Scale bar: 30 µm. (c) Mean cell polarizations, mean *β*, with respect to the direction of passing of the air bubble as a function of time (*green oval*). Control (*orange triangles*): cells exposed to continuous perfusion (*τ* = 0.14 dyn/cm^2^) without bubble. Error bars indicate SEM. (d) Establishment of flow-induced planar cell polarity in endothelial monolayer. Rearward mechanical displacement of nuclei under a direct action of hydrodynamic drag results in consistent polarization of confluent ECs against the flow.

To test the capacity of a simple mechanical force to establish polarization, we used a modified microfluidic device in which air bubbles were passed through a flow chamber with a confluent monolayer of non-polarized HUVECs (Fig. 4a). The passage of the front edge of a slowly moving air bubble caused nearly instantaneous translocations of the nuclei downstream (Fig. 4b). Apparently, the passage of the bubble front generated large short-term hydrodynamic drag on the nucleus, leading to rapid rearrangement of the cytoskeleton and inducing cell signaling response (Sobolewski et al., 2011). The large magnitude (8 µm on average) and short time scale (<5 sec) of the nucleus translocations makes it unlikely that the translocations are caused by some active intracellular transport (Gomes et al., 2005; Lee et al., 2005). The downstream displacement of the nuclei resulted in polarization of HUVECs against the direction of passage of the bubble (*β*>0; Fig. 4c; supplementary material Movie 5). Similar to the shear flow experiments (Fig. 1d), the polarization persisted for at least 1 hr after the passage of the bubble, while cells were exposed to a near-zero shear (*τ* = 0.14 dyn/cm^2^, far too low to cause polarization; Fig. 4c).

## Discussion

In summary, our data indicate that the nucleus of an EC can serve as a sensor of the strength and direction of blood flow. The nucleus is the largest and most resistant to compression organelle in the cell and has been previously suggested to have a capacity to act as a shock absorber (Dahl et al., 2004). Our results indicate that the nuclei of ECs can be displaced downstream under a direct action of hydrodynamic drag. The acto-myosin cytoskeleton resists the hydrodynamic drag applied to the nucleus, thereby controlling the sensitivity of polarization response of ECs to shear flow. Therefore, confluence- and flow-dependent rearrangements of the acto-myosin cytoskeleton can influence EC polarization not only through the active transport of organelles but also through changes in the passive resistance to the displacement of nucleus under the action of hydrodynamic shear. The downstream displacement of the nucleus directly contributes to the establishment of planar cell polarity in endothelium (Fig. 4d) and may also trigger cell signaling events eventually leading to relocation of MTOC by cytoskeletal motors (McCue et al., 2006; Morgan et al., 2011; Tzima et al., 2003).

A previously described mechanosensory complex responsible for sensing of hydrodynamic shear by ECs is located at cell–cell adhesions leads to integrin activation (Tzima et al., 2005); however, this complex does not appear to sense the directionality of the flow. As a result of shear stress experienced by the cellular membrane, cell–cell adhesions at different locations at the cell periphery are subjected to stretching forces that have different directions with respect to the cell boundary. The mechanosensory complex is activated by these forces triggering cytoskeleton remodeling, leading to elongation of ECs. In addition to adjacent cells, the mechanosensory complex is mechanically linked to the nuclear envelope through actin cytoskeleton (Leckband et al., 2011; Parsons et al., 2010). Displacement of the nucleus is thus expected to result in tensile forces on nuclear envelope-linked actin cytoskeleton and, consequently, on cell–cell adhesions, possibly triggering a mechano-sensing response. A Nesprin-mediated linkage between the nuclear envelope and the acto-myosin cytoskeleton is necessary for polarization of confluent ECs in response to shear flow (Morgan et al., 2011). Therefore, we propose that the tension resulting from hydrodynamic drag applied to EC nuclei provides a directional bias in the stretching forces in the mechanosensory complex at the cell–cell adhesions, thus facilitating cellular polarization under shear.

## Materials and Methods

### Constructs

H2b–EGFP and H2b–mCherry in SIN18.hPGK.eGFP.WPRE lentiviral vector were gifts from J. H. Price (Kita-Matsuo et al., 2009). GFP–α-tubulin in pRRL.PPT.CMV lentiviral vector was kindly provided by O. Pertz. Lentiviruses were produced as described (Kita-Matsuo et al., 2009). P23-YFP construct was a gift from R. Duden.

### Reagents

Blebbistatin and latrunculin A were from Enzo Life Sciences (Farmingdale, NY). Alexa-488 conjugated phalloidin was from Invitrogen (Carlsbad, CA).

### Microfluidic devices

Microfluidic devices were fabricated and used as previously described (Tkachenko et al., 2009).

### Culturing and imaging of ECs in vitro

Culturing of HUVECs (Lonza, Basel, Switzerland) was done as described (Tkachenko et al., 2009). Prior to imaging, cells were plated on fibronectin coated glass coverslips. Application of microfluidic devices was done as described (Tkachenko et al., 2009). Live microscopy was done in an environmentally controlled microscopy system (Tkachenko et al., 2009; Tkachenko et al., 2011).

### Super-high resolution microscopy imaging

Super-high resolution microscopy imaging was done using DeltaVision OMX system from Applied Precision (WA, USA).

### Imaging of ECs in mouse vessels

Male C57Bl/6 mice (10 w old) were anaesthetized and perfused at 100 mm Hg with saline for 2 min followed by 3.7% formalin/0.005% glutaraldehyde in PBS for 3 min. Vessels were harvested and, for aortas, adventitias were removed. The vessels were mounted en face on poly-L-Lysine-coated microscope slides by incubating the vessels for 5 min with 3.7% formalin under a coverslip. Next, the vessels were pre-treated with 0.5% Triton X-100 in PBS (5 min) and 1% BSA in PBS (1 h), followed by incubation with the primary antibodies over night at 4°C and secondary antibodies for 2 h at RT. The following antibodies were used: goat anti-mouse CD31 (1:400, R&D Systems, cat NO. AF3628), rabbit anti-human 58K Golgi protein (1:200, Abcam, cat NO. ab5820), PerCP-conjugated donkey anti-goat (1:50, Jackson ImmunoResearch, cat NO. 705-126-142) and Cy3-conjugated donkey anti-rabbit Cy3 (1:250, Jackson ImmunoResearch, cat NO. 711-116-152). The slides were rinsed in PBS between each step and mounted with ProLong® Gold antifade medium with DAPI (Invitrogen). Imaging of mouse vessels were taken under a fluorescence microscope (Zeiss, Axioplan 2) with 20× objective.

### Planar polarization analysis of ECs

Planar polarization analysis of ECs was done using ImagePro 6.1 (MediaCybernatics, Bethesda, MD) and home-build applications in Matlab (Mathworks, Natick, MA).

## Supplementary Material

Supplementary Material
