# Supplementary material for: The nucleus of endothelial cell as a sensor of blood flow direction
Source: Biol Open. 2013 Aug 14;2(10):1007–12. doi: 10.1242/bio.20134622 (PMC3798183; doi:10.1242/bio.20134622)
Supplement: Supplementary Material [file supp_2_10_1007__index.html]

The nucleus of endothelial cell as a sensor of blood flow direction — The nucleus of endothelial cell as a sensor of blood flow direction — Supplementary Material 

# The nucleus of endothelial cell as a sensor of blood flow direction

## bio.20134622 Supplementary Material

**Files in this Data Supplement:**

- Supplementary Material - Eugene Tkachenko et al. doi: 10.1242/bio.20134622
- Movie 1 - **Movie 1. Flow-induced changes in planar polarization of ECs as indicated by alignment of the Golgi and the nucleus relative to the flow.** Changes in the relative positioning of the Golgi and the nucleus in HUVECs upon exposure to flow shear stress *τ*=14.5 dyn/cm2 were monitored for 300 min. Direction of the flow is from left to right. Left side is an inverted fluorescent image (*grey*) showing an expression of the Golgi marker p23-eGFP. Right side is a brightfield image overlaid with vectors indicating positions of the Golgi relative to cell nuclei. Color-code indicates planar polarization relative to the direction of flow from *blue* (*β*=−90°; along the flow) to *red* (*β*=90°; against the flow). Images were acquired every 5 min. Frame rate is 6 per sec. Scale bar: 20 µm.
- Movie 2 - **Movie 2. Flow-induced changes in planar polarization of ECs as indicated by alignment of the MTOC and the nucleus relative to the flow.** Changes in the relative positioning of the MTOC and the nucleus in HUVECs upon exposure to flow shear stress *τ*=14.5 dyn/cm2 were monitored for 300 min. Inverted fluorescent images (*grey*) show an expression of GFP–α-tubulin. *Black arrow* shows the direction of flow. Images were acquired every 10 min. Frame rate is 3 per sec. Scale bar: 30 µm.
- Movie 3 - **Movie 3. Flow-induced changes in planar polarization of EC after treatment with latrunculin A.** Rapid change of the relative positioning of the MTOC (*green*) and the nucleus in HUVEC exposed to shear stress *τ*=7.2 dyn/cm2 after transient depolymerization of F-actin due to the treatment with 1 µM latrunculin A for 15 min. Brightfield images are depicted in *grey*. Direction of the flow is from up to down. Images were acquired every 5 min. Frame rate is 2 per sec.
- Movie 4 - **Movie 4. Flow-induced changes in planar polarization of EC after treatment with blebbistatin.** Rapid change of the relative positioning of the MTOC (*grey*) and the nucleus in HUVEC exposed to shear stress *τ*=7.2 dyn/cm2 after inhibition of myosin II due to the treatment with 30 µM blebbistatin for 30 min. Direction of the flow is from up to down. Images were acquired every 10 min. Frame rate is 2 per sec.
- Movie 5 - **Movie 5. Mechanical displacement of the nucleus by passing air bubble shifts polarization of ECs towards the direction of applied force.** Planar polarization of HUVECs based on the relative positioning of the MTOC and the nucleus was measured before and after passing an air bubble. An air bubble was moving along the flow from left to right. Fluorescent images (*grey*) show an expression of GFP–α-tubulin. Insert contains a color bar and an arrow indicating average planar polarization relative to the direction of passing air bubble for each time frame. Color-coded vectors correspond to planar polarization of individual cells from *blue* (*β*=−90°; along the flow and the direction of passing air bubble) to *red* (*β*=90°; against the flow). The front edge of the passing air bubble is seen at time point 20 min. The air bubble left the viewing area between time points 60 and 65 min. Note that the dragging force applied by passage of the air bubble caused an about 15° shift in average planar polarization angle toward the applied force and this angle remained unchanged for the rest of an experiment. Images were acquired 5 min apart. Frame rate is 1 per sec. Scale bar: 50 µm.
